# Brain Tumor Classification and Segmentation in MR Images Using EfficientNet and U-Net++ Models

**DOI:** 10.3390/diagnostics16111745

**Published:** 2026-06-05

**Authors:** Reema Alkharaan, Jana Alobaidi, Joud Bakarman, Hala Alshamlan

**Affiliations:** Department of Information Technology, College of Computer and Information Sciences, King Saud University, P.O. Box 51178, Riyadh 11543, Saudi Arabia; 443200466@student.ksu.edu.sa (R.A.); 444200842@student.ksu.edu.sa (J.A.); 443201057@student.ksu.edu.sa (J.B.)

**Keywords:** brain tumor, magnetic resonance imaging, deep learning, classification, segmentation

## Abstract

**Background/Objectives:** Brain tumor analysis using magnetic resonance imaging (MRI) remains a challenging task due to tumor heterogeneity, complex anatomical structures, and reliance on expert interpretation. Although deep learning approaches have shown promising results in medical image analysis, many existing studies focus on either tumor classification or segmentation independently, limiting their applicability in comprehensive automated brain tumor analysis workflows. This study proposes an integrated dual-task deep learning framework for automated brain tumor classification and segmentation using MRI scans. The framework aims to provide complementary diagnostic support by combining tumor-type prediction and tumor boundary delineation within an integrated workflow. **Methods:** The proposed framework utilizes EfficientNet-based convolutional neural networks for multi-class brain tumor classification and U-Net++ architectures with EfficientNet encoders for tumor segmentation. Experiments were conducted using the BRISC2025 dataset, consisting primarily of 6000 T1-weighted 2D MRI slices collected from axial, coronal, and sagittal planes. Standard preprocessing, augmentation, transfer learning, and selective fine-tuning strategies were applied. Multiple architectures were systematically evaluated using evaluation metrics. **Results:** EfficientNet-B1 achieved a classification accuracy of 99.70% with near-perfect precision, recall, and F1-scores across glioma, meningioma, pituitary tumor, and no-tumor classes. For segmentation, U-Net++ with an EfficientNet-B1 encoder achieved a Dice score of 0.9055, an IoU score of 0.8442, and an HD95 value of 12.21 pixels on the held-out test set. The proposed framework demonstrated robust performance in detecting small and low-contrast tumor regions while maintaining strong generalization performance across diverse MRI samples. **Conclusions:** The proposed integrated framework demonstrated strong performance in both brain tumor classification and segmentation tasks, effectively detecting small and low-contrast tumor regions while maintaining good generalization across diverse MRI samples. These findings suggest that the framework may serve as a reliable decision-support tool for automated brain tumor analysis in clinical practice.

## 1. Introduction

Brain tumors represent a serious neurological condition that can significantly affect cognitive functions, motor abilities, and overall quality of life. Accurate diagnosis plays a critical role in treatment planning and patient outcomes, particularly given the diverse characteristics of brain tumors and their varying growth patterns. Magnetic resonance imaging (MRI) is widely used as a primary imaging modality for brain tumor assessment due to its high spatial resolution and ability to capture detailed soft tissue information. However, interpreting MRI scans remains a challenging and time-consuming task that requires expert knowledge and is subject to inter-observer variability among radiologists.

Recent advances in artificial intelligence (AI), particularly deep learning (DL), have shown promising potential in addressing challenges within medical image analysis. Deep learning models based on convolutional neural networks (CNNs) have been extensively applied to MRI-based brain tumor analysis, achieving notable performance in tasks such as tumor classification and segmentation.

Several studies have focused on classifying brain tumors into different categories, while others have concentrated on segmenting tumor regions to delineate tumor boundaries precisely. Although high accuracy has been reported in many cases, the performance of these approaches often depends on dataset quality and model robustness. Many existing studies address classification or segmentation as isolated tasks, which may limit their applicability in clinical workflows.

In this study, we propose an integrated dual-task framework for automated brain tumor classification and segmentation using MRI scans. The framework employs CNN architectures to analyze MRI data and generate both classification predictions and segmentation outputs. The approach is evaluated using a multi-source MRI dataset to assess performance across tumor types and imaging conditions. Results demonstrate promising performance in both classification and segmentation tasks, highlighting the potential of deep learning frameworks in advancing brain tumor analysis.

### Contributions

The main contributions of this work are as follows:

• Development of an integrated dual-task framework for brain tumor classification and segmentation.
• Comprehensive evaluation of multiple state-of-the-art classification and segmentation architectures.
• Investigation of transfer learning and selective fine-tuning strategies using EfficientNet backbones.
• Demonstration of strong classification and segmentation performance on multi-source MRI datasets.
• Analysis of model robustness through quantitative and qualitative evaluation.

## 2. Background

Brain tumors are among the most complex neurological conditions due to their direct impact on essential brain functions and patient quality of life. Heterogeneity in tumor location, size, shape, and growth behavior makes accurate diagnosis particularly challenging. Early and reliable detection is critical for effective treatment planning, surgical intervention, and disease monitoring, highlighting the need for robust and automated MRI-based analysis approaches.

### 2.1. Brain Tumor Diagnosis and MRI Challenges

MRI provides high-resolution and superior soft-tissue contrast, allowing clinicians to visualize tumor boundaries, internal structures, and surrounding brain tissues. Despite its advantages, MRI interpretation is complex and time-consuming, relying heavily on expert radiological judgment. Tumor appearance varies across patients and imaging protocols, and subtle differences between tumor tissue and healthy structures may complicate visual assessment. Inter-observer variability can lead to diagnostic inconsistencies, motivating automated analysis systems.

Tumor types such as gliomas, meningiomas, and pituitary tumors present additional diagnostic challenges due to differences in morphology, growth patterns, and MRI appearance, reinforcing the need for automated, robust segmentation and classification methods.

### 2.2. Deep Learning for Brain Tumor Classification and Segmentation

Deep learning enables models to learn hierarchical feature representations directly from data, without relying on handcrafted features. CNNs are particularly effective for visual analysis tasks in medical imaging. Brain tumor analysis commonly involves:

• Classification: Determining tumor presence and type (e.g., glioma, meningioma, pituitary). CNNs extract semantic features from MRI scans to differentiate tumor types.
• Segmentation: Delineating tumor regions at the pixel level. Segmentation is critical for estimating size, defining boundaries, surgical planning, and monitoring disease progression.

Metrics such as the Dice similarity coefficient (DSC), Intersection over Union (IoU) and Hausdorff Distance-based are used to quantify segmentation performance.

## 3. Literature Review

In this section, we will discuss recent studies on brain tumor detection, showcasing how research on brain tumor detection has grown rapidly over the past decade, driven by the need for accurate, fast, and reliable diagnostic support. Most studies focus on two main tasks: classification and segmentation, yet challenges remain in terms of model interpretability, clinical integration, and real-world usability.

From 2019 to 2025, research on brain tumor detection and segmentation using MRI has advanced rapidly, with studies exploring different approaches to improve accuracy and efficiency.

In January 2019, Swati et al. [1] explored brain tumor classification using the FigShare Brain MRI Dataset [2], which also forms part of our dataset. They employed pretrained CNN architectures through transfer learning and reported a top performance of Prec@10 = 94.39%. For feature extraction, they relied on the CNN layers to automatically learn image representations. Additionally, a CBIR (Content-Based Image Retrieval) system for brain tumor retrieval used a pretrained VGG19 model with block-wise fine-tuning, extracting features from the Fully Connected layer 7 and applying metric learning for similarity matching. However, Swati et al.’s study was limited to classification and did not include segmentation, reducing its broader diagnostic applicability.

By March 2019, Gumaei et al. [3] investigated brain tumor classification using the FigShare Brain MRI Dataset [2]. They introduced a Regularized Extreme Learning Machine (RELM), which achieved an accuracy of 92.61%. For feature extraction, they used a hybrid approach: handcrafted features (texture and shape) were extracted using Gray Level Co-occurrence Matrix (GLCM) and Histogram of Oriented Gradients (HOG), while deep features were obtained from a pretrained CNN. These two feature sets were fused into a comprehensive representation, which was then fed into the RELM classifier for final tumor classification. Similar to earlier work, their study focused only on classification and did not attempt segmentation, limiting its overall diagnostic scope.

In 2021, Micallef et al. [4] evaluated a U-Net++-based model for automatic brain tumor segmentation using the BraTS 2019 dataset [5]. Their approach applied standard data preprocessing and data augmentation techniques and relied on the nested skip-connection design of U-Net++ to extract multi-scale tumor features. The model achieved Dice scores of 0.7192 for Enhancing Tumor, 0.8712 for Whole Tumor, and 0.7817 for Tumor Core on the validation set, showing that U-Net++ can provide strong segmentation performance while remaining relatively lightweight.

In July 2022, Filatov et al. [6] examined brain tumor classification using the Brain Tumor MRI Dataset [7]. They compared several architectures, including ResNet50, EfficientNet-B1/B7, and EfficientNetV2B1, with EfficientNet-B1 achieving the highest accuracy of 89.6%. As for feature extraction, this study relied on pretrained ImageNet weights; the initial layers were frozen to preserve previously learned features, and these features were reused as the foundation for classification. However, their study did not address segmentation, which limited its applicability for comprehensive diagnostic support.

In 2023, Gómez-Guzmán et al. [8] explored brain tumor classification with convolutional neural networks, showing that CNN-based models remain effective for distinguishing tumor types. They evaluated six pretrained CNN/transfer learning models, with InceptionV3 achieving the highest classification accuracy of 97.12%. Feature extraction was handled within the internal convolutional layers of the pretrained CNN models through transfer learning. However, their work focused only on classification and did not attempt segmentation, limiting its practical diagnostic value.

In June 2024, Abdusalomov et al. [9] used Yolov7 for tumor classification across multiple datasets, achieving 99.5% accuracy. Feature extraction was handled within the layers of YOLOv7, and segmentation was not addressed in this study.

In September 2024, Poudyal et al. [10] used the Brain Tumor MRI Dataset to evaluate VGG16, ResNet50, DenseNet121, and InceptionV3, achieving the highest accuracy with DenseNet121 at 94%. The authors utilized the internal layers of the pretrained CNN models to automatically learn and extract relevant features from the MRI images. No segmentation was included.

By 2025, studies became even more ambitious. Gündogan [11] introduced a hybrid model combining CNN and XGBoost, reaching 99.8% accuracy, one of the highest reported. For feature extraction, the CNN was used to generate deep feature representations from MRI images by removing its final dense classification layer, and these extracted features were then passed into XGBoost for the final classification. In addition, Grad-CAM was applied to highlight the image regions most influential in the decision process, improving explainability alongside performance.

In June 2025, Xiong et al. [12] developed YOLO-BT, which stood out by performing both classification (mAP@50 of 94.7%) and segmentation (Dice score of 92.6%), though it was still limited to binary tumor detection (tumor, non-tumor) and did not classify the type of tumor. Feature extraction in YOLO-BT was performed through the UNetV2 backbone, which generated multi-scale feature maps and preserved spatial details via skip connections. The extraction process was further enhanced with attention mechanisms and the D-LKA module, improving the network’s ability to capture tumor boundaries and irregular shapes.

In July 2025, Valerio et al. [13] focused on segmentation using SegResNet, achieving a Dice score of about 0.90. They also incorporated LLMs to generate comprehensive, human-readable medical reports, enhancing explainability and interpretability, but the LLM was not integrated into the brain tumor diagnosis process itself. Classification was not addressed in this study.

The following month, in August 2025, Aksoy et al. [14] reported around 98.1% accuracy for classification and Dice scores between 0.68 and 0.70 for segmentation, though the segmentation results were notably weaker compared to more recent work. For classification, feature extraction was handled within the layers of MobileNetV2, while for segmentation, they manually extracted volumetric tumor subregion counts (Whole Tumor “WT”, Tumor Core “TC”, Enhancing Tumor “ET”) from the segmentation output.

Around the same time, in August 2025, Chen et al. [15] compared multiple architectures, with results ranging from 93.9%with ResNet101V2 to 99.79% with VGG19. This study highlights the varying performance of different architectures, with VGG19 achieving the highest accuracy, likely due to its deeper network facilitating the capture of more complex features. In contrast, ResNet101V2 exhibited the lowest accuracy, potentially due to its residual connections being less effective for this specific dataset. However, this study, too, was limited to classification alone.

In conclusion, the variations in reported accuracies across these studies stem from differences in datasets, model architectures, preprocessing strategies, and whether segmentation was incorporated alongside classification. Pretrained CNNs and transfer learning models, such as InceptionV3, VGG19, and EfficientNet, consistently achieved strong results by leveraging features learned from large-scale image datasets, making them particularly effective for distinguishing between tumor types. Hybrid approaches, such as CNN-XGBoost, enhanced robustness by integrating deep feature extraction with traditional machine learning. Object detection and segmentation-based models, including Yolov7, YOLO-BT, and SegResNet, were better suited for tumor localization and treatment planning, though their classification performance was sometimes lower than CNN-only methods. Meanwhile, studies incorporating LLMs or explainable AI techniques advanced interpretability and usability by generating human-readable reports, but these did not necessarily improve classification.

Despite the significant progress achieved by previous studies, several limitations remain. Most existing approaches focus on either classification or segmentation as independent tasks, which limits their ability to provide comprehensive diagnostic support. In addition, performance comparisons across studies are often challenging due to differences in datasets, preprocessing techniques, and evaluation protocols. Although some recent works attempted to combine multiple tasks, they were either limited to binary tumor detection or focused primarily on segmentation without providing multi-class tumor classification. Furthermore, the integration of classification and segmentation within a unified framework remains relatively underexplored in the literature. These limitations motivated the development of the proposed framework, which integrates multi-class brain tumor classification and tumor segmentation within a unified workflow for comprehensive brain tumor analysis.

Furthermore, since we are working with a diverse dataset, we will experiment with several of the highest-performing models reported in the literature such as EfficientNet, VGG19, U-Net++, SegResNet, and others, to identify the architectures that deliver the most stable and generalizable performance for both segmentation and classification tasks.

The following section presents a comparison of the mentioned academic studies on brain tumor detection. Table 1 summarizes their methods, datasets, and performance metrics to provide a clear overview of the current research landscape.

## 4. Materials and Methods

### 4.1. Data Preparation

#### 4.1.1. Dataset Description

The BRISC2025 dataset (Fateh et al., 2025 [16]) was used for both classification and segmentation tasks. BRISC2025 is a curated and expert-annotated brain tumor MRI dataset designed for multi-class classification and pixel-wise segmentation. The dataset contains 6000 T1-weighted MRI slices, including 5000 training images and 1000 testing images. The MRI slices were collected from three anatomical planes: axial, coronal, and sagittal.

For the classification task, the dataset contains four classes: glioma, meningioma, pituitary tumor, and no tumor. The original dataset was provided with predefined training and testing partitions. Table 2 summarizes the class distribution of the classification dataset.

For the segmentation task, the BRISC2025 dataset provides paired MRI images and corresponding expert-reviewed binary tumor masks. The segmentation subset consisted of 4793 annotated MRI slices, all of which were included within the larger 6000-image classification dataset. Each MRI image is associated with a pixel-wise segmentation mask representing the tumor region. The dataset contains aligned image–mask pairs, where each mask shares the same filename as its corresponding MRI image. Furthermore, all segmentation masks were reviewed and corrected by medical experts to ensure annotation quality and consistency. The segmentation dataset was subsequently divided into training, validation, and testing subsets as described in the following sections.

#### 4.1.2. Segmentation Data Preparation

To prepare the MRI images and corresponding segmentation masks for training and evaluation, several preprocessing steps were applied to the BRISC2025 dataset [16]. All preprocessing procedures were performed using Google Colab (Google LLC, Mountain View, CA, USA).

##### Data Splitting

The segmentation dataset consists of paired MRI images and ground-truth masks. Although the original dataset was provided with a predefined training–testing split, the data were reorganized using a hold-out partitioning strategy into 70% training, 15% validation, and 15% testing sets. Images and masks were stored in separate directories while preserving their one-to-one correspondence.

##### Image Resizing

All MRI images and their corresponding segmentation masks were resized to a fixed resolution of 512 × 512 pixels. Area interpolation was applied to the MRI images to preserve visual details, whereas nearest-neighbor interpolation was used for the segmentation masks to maintain discrete label boundaries.

##### Normalization

The MRI images were normalized using min–max scaling by dividing pixel intensity values by 255.0, resulting in values within the [0, 1] range. Segmentation masks were excluded from normalization, as they represent categorical pixel-wise labels.

##### Data Augmentation

To enhance model generalization, data augmentation was applied exclusively to the training set. Each image–mask pair was augmented jointly to preserve spatial alignment. The applied augmentations included random horizontal and vertical flipping, 90-degree rotations, brightness adjustments (applied to images only), shift–scale–rotate transformations, and Gaussian noise injection. Validation and test sets were left unchanged to ensure unbiased performance evaluation. A summary of the segmentation data preprocessing steps is presented in Table 3.

#### 4.1.3. Classification Data Preparation

To prepare the MRI images for the brain tumor classification task, several preprocessing steps were applied to the BRISC2025 classification dataset [16]. These steps were designed to ensure data consistency, enhance model compatibility, and improve training stability. All preprocessing operations were conducted using Google Colab.

The utilized dataset consisted primarily of T1-weighted MRI brain scans obtained from the BRISC2025 dataset. T1-weighted MRI images provide clear anatomical visualization of brain structures and are commonly used for brain tumor assessment and structural analysis. The study focused on 2D MRI image analysis using single-modality T1-weighted images and did not perform multimodal fusion or modality-specific comparison between different MRI sequences.

##### Data Cleaning

The initial preprocessing step involved converting all MRI images to the RGB color format. This step ensures uniformity across the dataset, as some images were originally stored in grayscale or different channel configurations. Converting all images to RGB allows seamless compatibility with the EfficientNet-B1 architecture, which expects three-channel input images.

##### Image Resizing

After data cleaning, all MRI images were resized to a fixed resolution of 224 × 224 pixels using area interpolation. This resolution was selected to match the input requirements of the EfficientNet-B1 model, ensuring efficient processing and consistent feature extraction during training.

##### Normalization

Following resizing, all MRI images were normalized using the standard ImageNet normalization parameters. Specifically, pixel values were normalized using predefined mean values [0.485, 0.456, 0.406] and standard deviation values [0.229, 0.224, 0.225] to ensure compatibility with the pretrained EfficientNet-B1 architecture. This normalization strategy improves numerical stability and facilitates effective transfer learning from ImageNet-pretrained weights.

##### Label Encoding

After normalization, categorical class labels were converted into numerical representations to facilitate supervised learning. The class-to-label mapping was defined as follows: glioma: 0, meningioma: 1, no tumor: 2, pituitary: 3. This encoding enables efficient loss computation and model optimization during training.

##### Data Splitting

In the final preprocessing step, the original BRISC2025 training subset was further divided into training and validation sets using an 80/20 split. The original testing subset provided by the dataset was kept completely isolated and was used only for final model evaluation. No images from the testing subset were used during training, validation, hyperparameter tuning, or model selection, reducing the risk of data leakage and ensuring an unbiased assessment of model performance. A summary of the classification data preprocessing steps is presented in Table 4.

### 4.2. Segmentation Model and Training Setup

The proposed segmentation framework is based on a U-Net++ architecture employing an EfficientNet-B1 encoder pretrained on ImageNet. The encoder serves as a hierarchical multi-scale feature extractor, enabling the model to capture both low-level spatial details and high-level semantic information relevant to brain tumor regions.

To balance feature reuse and task-specific adaptation, a selective fine-tuning strategy was adopted during training. Specifically, all EfficientNet-B1 encoder layers were frozen except the final encoder stage, corresponding to blocks.15–blocks.21, which remained trainable to adapt high-level semantic representations to tumor-specific segmentation features. The U-Net++ decoder and segmentation head were also kept trainable throughout training. No separate decoder warm-up stage was applied; partial fine-tuning was performed from the beginning of training. The reported encoder block indices follow the internal layer organization of the PyTorch (version 2.5.1)-based segmentation framework used in this study [18].

Model optimization was performed using the Adam optimizer with a CosineAnnealingWarmRestarts learning rate scheduler to dynamically adjust the learning rate during training. The network was trained for up to 60 epochs, with early stopping applied based on validation Dice score to mitigate overfitting. Segmentation performance was evaluated on both validation and test sets using standard overlap-based metrics.

### 4.3. Segmentation Model Loss Functions and Evaluation Metrics

To address class imbalance and improve boundary delineation, a composite loss function combining Dice Loss and Focal Loss was employed. The total segmentation loss is defined as follows:

$$L_{total}=0.7\cdot L_{Dice}+0.3\cdot L_{Focal}$$

#### 4.3.1. Dice Coefficient and Dice Loss

The Dice coefficient measures the overlap between the predicted segmentation P and the ground-truth mask G, and is defined as follows:

$$\mathrm{Dice}=\frac{2TP}{2TP+FP+FN}$$

where TP, FP, and FN denote the number of true positives, false positives, and false negatives, respectively. Dice Loss is derived directly from the Dice coefficient as follows:

$$L_{Dice}=1-\mathrm{Dice}$$

#### 4.3.2. Focal Loss

Focal Loss was incorporated to reduce the impact of easily classified background pixels and to focus learning on hard-to-classify tumor regions. It is defined as follows:

$$L_{Focal}=-\alpha (1-p_{t}{)}^{\gamma }log(p_{t})$$

where $p_{t}$ represents the predicted probability of the true class, and α and γ are hyperparameters that control class weighting and focusing strength, respectively.

#### 4.3.3. Segmentation Evaluation Metrics

Segmentation performance was quantitatively assessed using the Dice coefficient, Intersection over Union (IoU) and the 95% Hausdorff Distance (HD95). Unlike the standard Hausdorff Distance, HD95 reduces sensitivity to extreme outlier points by considering the 95th percentile of the boundary distances.

IoU is defined as follows:

$$\mathrm{IoU}=\frac{TP}{TP+FP+FN}$$

These metrics provide complementary perspectives on segmentation accuracy and spatial overlap between predicted tumor regions and ground-truth annotations.

### 4.4. Classification Model and Training Setup

The proposed classification framework is built upon the EfficientNet-B1 architecture pretrained on the ImageNet dataset. EfficientNet-B1 is adopted as a feature extraction backbone due to its strong capability to learn hierarchical visual representations, capturing both low-level features (e.g., edges and textures) and high-level semantic patterns relevant to brain tumor characterization in MRI images.

To balance feature reuse and task-specific adaptation, a selective fine-tuning strategy was employed during training. Specifically, only the first EfficientNet-B1 block (blocks.0) was frozen to preserve early low-level ImageNet-pretrained representations. The remaining EfficientNet-B1 blocks, together with the newly replaced classification head, were kept trainable throughout the training process to learn tumor-specific discriminative features. No separate warm-up stage was applied for the classifier; fine-tuning was performed from the beginning of training using the trainable layers.

Model optimization was conducted using the Adam optimizer with a learning rate of 1 × 10^−4^, ensuring stable and efficient convergence during fine-tuning. The network was trained for a maximum of 50 epochs, with Early Stopping applied based on validation accuracy to prevent overfitting. Additionally, a learning rate scheduler was utilized to dynamically adjust the learning rate when validation performance plateaued. Model performance was evaluated on both validation and test sets using standard multi-class evaluation metrics.

### 4.5. Loss Function and Evaluation Metrics

#### 4.5.1. Classification Loss Function

For the multi-class brain tumor classification task, the cross-entropy loss function was employed. This loss function is widely used in multi-class classification problems, as it quantifies the discrepancy between the predicted class probability distribution and the ground-truth labels.

The cross-entropy loss is defined as follows:

$$L_{CE}=-\sum_{i=1}^{C}y_{i}\log\left({\hat{y}}_{i}\right)$$

where *C* denotes the number of classes, *y_i_* represents the ground-truth label for class $i^{y}$, and *i* corresponds to the predicted probability of class.

#### 4.5.2. Classification Evaluation Metrics

To comprehensively assess the performance of the proposed EfficientNet-B1 classification model, several standard evaluation metrics were employed, including accuracy, precision, recall, and F1-score. These metrics are commonly used to evaluate multi-class classification systems in medical imaging applications.

True positive (TP) denotes tumor images correctly classified as tumorous, while true negative (TN) represents non-tumorous images correctly identified. False positive (FP) refers to non-tumorous images incorrectly classified as tumors, and false negative (FN) indicates tumor images misclassified as non-tumorous. Based on these definitions, the evaluation metrics are computed as follows:

$$Precision=\frac{TP}{FP+TP}$$

$$Recall=\frac{TP}{FN+TP}$$

$$F1-score=\frac{\left(\mathrm{Pr}ecision\times Recall\right)\times 2}{\mathrm{Pr}ecision+Recall}$$

$$Accuracy=\frac{TP+TN}{TP+TN+FP+FN}$$

## 5. Results

This section presents the experimental results and performance analysis of the proposed classification and segmentation frameworks. Quantitative evaluation metrics, comparative experiments, and visual analyses are provided to demonstrate the effectiveness and robustness of the model.

### 5.1. Segmentation Model Results

#### 5.1.1. Comparison of Segmentation Architectures

To identify the most suitable segmentation architecture for brain tumor segmentation, several deep learning architectures reported in the literature were implemented and systematically evaluated. All models were trained and tested using the same curated MRI dataset, identical preprocessing procedures, consistent train–validation–test splits, and unified evaluation metrics to ensure a fair and unbiased comparison.

The evaluated architectures include ResUNet, EfficientNet-B4, EfficientNet-B4 U-Net, Attention U-Net, U-Net 2D and U-Net++. Their performance in terms of training Dice, validation Dice, training IoU, and validation IoU is summarized in Table 5.

U-Net++ achieved the highest validation Dice score and was selected as the final segmentation architecture.

#### 5.1.2. Frozen Encoder Performance

To establish a baseline, all encoder layers were frozen and only the decoder and segmentation head were trained. The performance of frozen EfficientNet encoders is summarized below in Table 6.

Although both encoders performed well, EfficientNet-B1 demonstrated slightly higher Dice and IoU, with stable convergence.

#### 5.1.3. Partial Fine-Tuning Performance

Partial fine-tuning was applied by unfreezing only the deepest encoder blocks to enhance tumor-specific feature learning while preserving low-level features from ImageNet pretraining. The performance of the fine-tuned EfficientNet encoders is summarized below in Table 7.

The results indicate that partial fine-tuning enhances tumor-specific feature learning while preserving low-level features from ImageNet pretraining.

#### 5.1.4. Test Set Performance

Testing was conducted on the held-out test set using the best checkpoints for both encoders. Table 8 shows the testing performance.

The experiments presented in Table 6, Table 7 and Table 8 provide an ablation analysis of the proposed segmentation framework. Different encoder variants and training strategies were systematically evaluated to investigate their impact on segmentation performance. The results demonstrate that EfficientNet-B1 combined with partial fine-tuning consistently achieved superior Dice and IoU scores compared with the alternative configurations. The selected loss weighting (0.7 Dice + 0.3 Focal) was adopted based on empirical performance observed during preliminary experiments. A comprehensive evaluation of alternative loss–weight combinations remains an important direction for future work.

**Figure 1:**
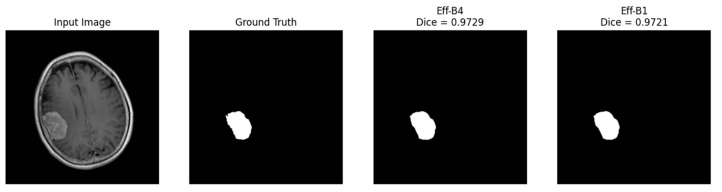
Representative segmentation example in which both EfficientNet-B1 and EfficientNet-B4 achieved high agreement with the ground-truth mask, producing accurate tumor localization and boundary delineation (Dice scores: 0.9721 and 0.9729, respectively).

**Figure 2:**
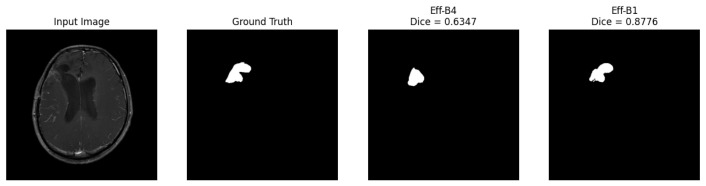
Segmentation example demonstrating improved tumor boundary delineation by EfficientNet-B1 compared with EfficientNet-B4. EfficientNet-B4 exhibited slight under-segmentation, resulting in a lower Dice score (0.6347) than EfficientNet-B1 (0.8776).

**Figure 3:**
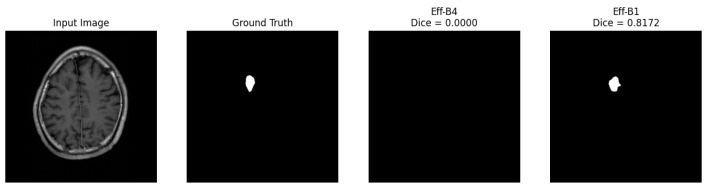
Challenging small-tumor case in which EfficientNet-B1 successfully detected the lesion and generated a segmentation mask closely matching the ground-truth annotation, whereas EfficientNet-B4 failed to identify the tumor region (Dice = 0.0000).

EfficientNet-B1 consistently outperformed B4 across all metrics, providing more accurate tumor boundary delineation, including small and low-contrast tumors. Figure 1, Figure 2 and Figure 3 illustrate representative examples of segmentation results; they display original test images, ground-truth masks, and predicted masks.

The proposed model, U-Net++ with EfficientNet-B1 and partial fine-tuning, achieved the highest Dice and IoU scores across all evaluations, demonstrating superior accuracy and robustness for brain tumor segmentation.

### 5.2. Classification Model Results

#### 5.2.1. Comparison of Classification Architectures

To identify the most suitable architecture for brain tumor classification, several deep learning and hybrid Architectures reported in the literature were implemented and evaluated using the same MRI dataset, identical preprocessing steps, consistent train–validation–test splits, and unified evaluation metrics to ensure fair comparison.

The evaluated architectures include VGG19, Extreme Learning Machine (ELM), a hybrid EfficientNetV2B3 + XGBoost classifier, EfficientNet-B4, and EfficientNet-B1. Their performance in terms of training accuracy, validation accuracy, training loss, and validation loss is summarized in Table 9.

Among all evaluated architectures, EfficientNet-B1 demonstrated the most consistent and superior performance across all evaluation metrics, making it the most suitable backbone.

#### 5.2.2. Overall Accuracy of EfficientNet-B1

The final test accuracy achieved by EfficientNet-B1 was 99.70%, indicating the model successfully learned meaningful tumor-specific features and can reliably classify unseen MRI scans.

#### 5.2.3. Class-Wise Classification Performance

All four classes—glioma, meningioma, pituitary tumor, and no tumor—achieved near-perfect performance. As shown in Table 10.

These results demonstrate that the classifier maintains consistent reliability across all categories without bias toward any specific class.

#### 5.2.4. Comparison with State-of-the-Art Classification Methods

To further evaluate the effectiveness of the proposed classification model, its performance was compared with several recently published state-of-the-art studies. As shown in Table 11, the proposed EfficientNet-B1 model achieved a classification accuracy of 99.70%, outperforming most previously reported methods and demonstrating competitive performance relative to the highest reported accuracy of 99.77%.

As shown in Table 12, the proposed U-Net++ model with an EfficientNet-B1 encoder achieved a Dice score of 0.9055, surpassing previously reported segmentation approaches. These results demonstrate the effectiveness of the proposed framework in accurately delineating brain tumor regions while maintaining robust generalization performance.

#### 5.2.5. Confusion Matrix Analysis

**Figure 4:**
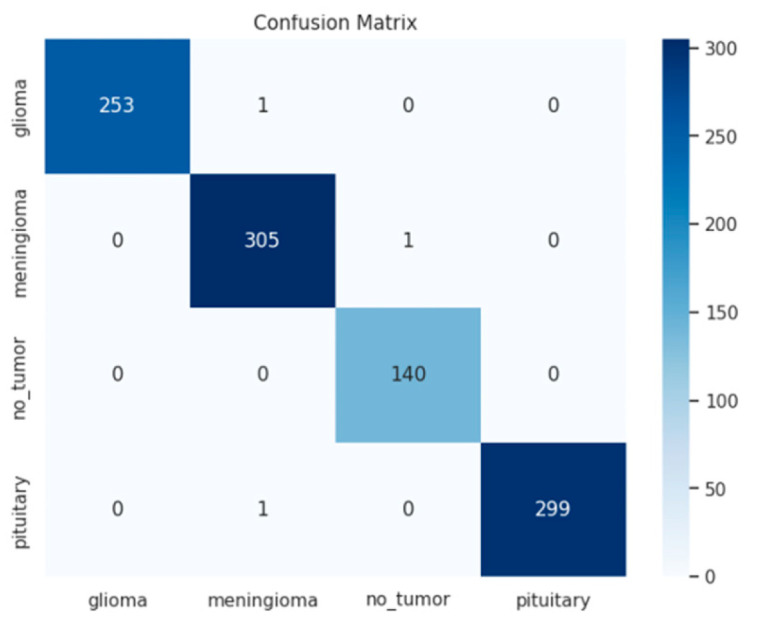
Confusion matrix of the EfficientNet-B1 classification model on the test set. The model achieved near-perfect classification performance, with only three misclassified cases among 1000 test images.

Figure 4 illustrates the confusion matrix of the EfficientNet-B1 classification model. The matrix demonstrates strong classification stability, with 997 out of 1000 MRI images correctly classified. Only three misclassification cases were observed:

1 case: glioma → meningioma
1 case: meningioma → no tumor
1 case: pituitary → meningioma

The extremely low misclassification rate highlights the model’s ability to distinguish subtle visual differences among various tumor types.

#### 5.2.6. Training Dynamics Analysis

##### Accuracy Curves

**Figure 5:**
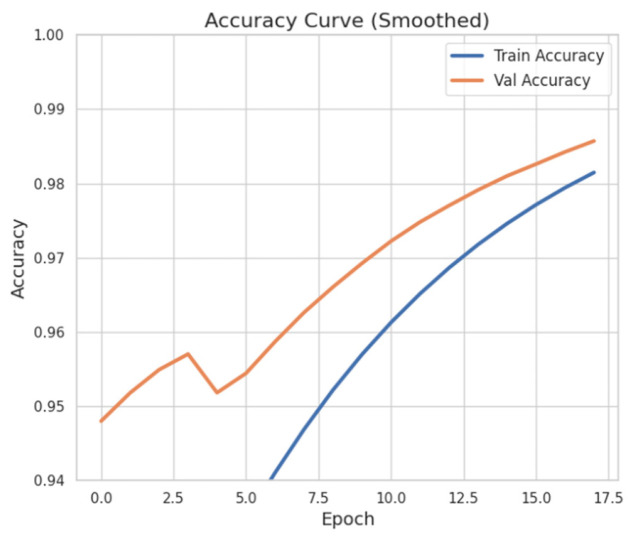
Training and validation accuracy curves of the EfficientNet-B1 classification model. Both curves demonstrate stable convergence and strong generalization performance, with validation accuracy closely matching training accuracy throughout the training process.

Figure 5 shows training and validation accuracy curves. Both curves exhibit a smooth upward trend with near-perfect convergence. Validation accuracy closely matches or slightly exceeds training accuracy, indicating absence of overfitting and strong generalization.

##### Loss Curves

**Figure 6:**
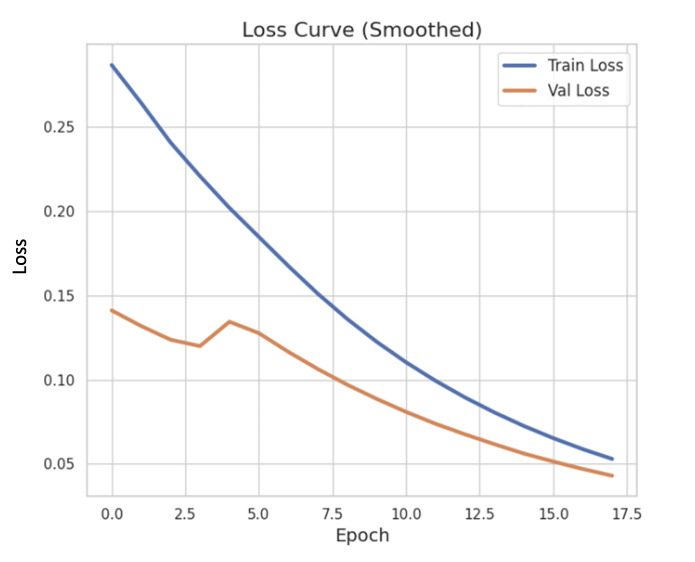
Training and validation loss curves of the EfficientNet-B1 classification model. The gradual reduction in loss values indicates stable optimization and effective learning, while the close alignment between training and validation loss suggests minimal overfitting.

Figure 6 shows the training and validation loss curves. A synchronized decrease is observed throughout training, reflecting stable optimization, effective learning rate scheduling, and successful early stopping. The final validation loss converged to approximately 0.04, further supporting the robustness and effectiveness of the proposed model.

#### 5.2.7. Grad-CAM Visualization Analysis

**Figure 7:**
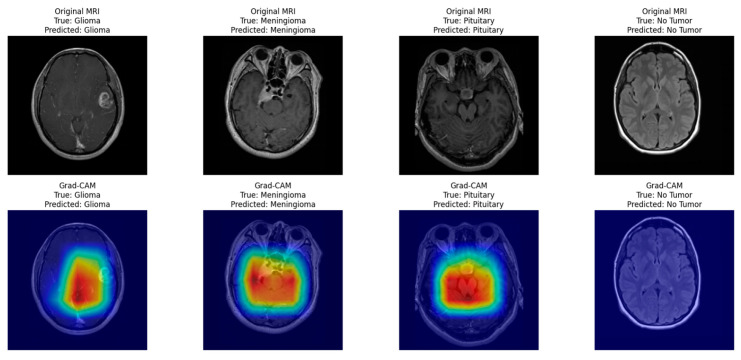
Grad-CAM visualizations for representative MRI test samples generated using the EfficientNet-B1 classification model.

To further investigate the interpretability of the proposed classification model, Grad-CAM visualizations were generated using the EfficientNet-B1 classifier for representative MRI test samples from the four classes: glioma, meningioma, pituitary tumor, and no tumor. Grad-CAM was employed as a post hoc explainability technique to highlight the image regions that contributed most strongly to the classification decisions of the model. Figure 7 presents both the original MRI images and their corresponding Grad-CAM heatmaps, along with the true labels and predicted labels generated by the classifier. The selected samples were all correctly classified by the EfficientNet-B1 model. In the tumor classes, the generated heatmaps demonstrated stronger activations around clinically relevant abnormal regions associated with tumor presence. In contrast, the no tumor sample exhibited comparatively low and diffuse activations without concentrated attention on suspicious regions, which is consistent with the absence of visible tumor abnormalities. These visual explanations suggest that the classification model learned meaningful tumor-related visual patterns rather than relying solely on irrelevant background information. Although Grad-CAM does not provide precise pixel-level localization comparable to dedicated segmentation models, it improves the interpretability of the classification process by illustrating the regions that most influenced the model’s predictions. This additional explainability component helps reduce the black-box nature commonly associated with deep learning-based medical image classification systems.

## 6. Discussion

The experimental results demonstrated that EfficientNet-B1 and U-Net++ with an EfficientNet-B1 encoder achieved the strongest overall performance among the evaluated architectures for classification and segmentation tasks. In the classification experiments, EfficientNet-B1 achieved a test accuracy of 99.70%, outperforming VGG19, ELM, EfficientNetV2B3 + XGBoost, and EfficientNet-B4 on the same dataset and under the same preprocessing and evaluation settings. The confusion matrix further showed that only three MRI images were misclassified among the 1000 test images, indicating consistent classification performance across all tumor classes.

For segmentation, U-Net++ achieved the highest validation Dice score among all evaluated segmentation architectures and was therefore selected as the final segmentation model. The final U-Net++ model with an EfficientNet-B1 encoder achieved a Dice score of 0.9055 and an IoU score of 0.8442 on the held-out test set. Representative segmentation examples also showed that EfficientNet-B1 produced segmentation masks with closer agreement to the ground-truth masks in several challenging cases, including small tumor regions.

The experiments also showed that partial fine-tuning achieved higher Dice and IoU scores compared with fully frozen encoders for both EfficientNet-B1 and EfficientNet-B4. These results indicate that partial fine-tuning improved segmentation performance under the experimental setup used in this study.

When compared with previously published studies, the proposed framework achieved competitive results for both classification and segmentation tasks. The EfficientNet-B1 classification model achieved higher accuracy than several previously reported methods, including those of Filatov et al., Gómez-Guzmán et al., and Poudyal et al., while achieving performance comparable to recent high-performing studies such as Gündogan and Chen et al. Similarly, the proposed U-Net++ segmentation model achieved a Dice score slightly higher than the SegResNet model reported by Valerio et al.

Although the proposed framework achieved strong performance, the experiments were conducted using a fixed train–validation–test split and a single experimental run. Future work will include repeated independent runs, statistical significance testing, and reporting results as mean ± standard deviation to further evaluate model stability, robustness, and reproducibility.

From a clinical perspective, the proposed framework has the potential to support radiologists by providing automated tumor classification and segmentation results as a decision-support tool. The classification model can assist in identifying tumor categories from MRI scans, while the segmentation model can provide accurate delineation of tumor boundaries, which may facilitate treatment planning and disease monitoring. By integrating both tasks within a unified workflow, the framework can help reduce manual workload and improve diagnostic efficiency. Nevertheless, the proposed system is intended to assist clinical decision-making rather than replace professional medical judgment, and further validation in real clinical environments is required before deployment in healthcare settings.

## 7. Conclusions

In this study, we explored the use of deep learning for brain tumor classification and segmentation using MRI data. Brain tumor diagnosis remains a challenging clinical task due to tumor heterogeneity, subtle differences across classes, and variable image quality.

For tumor classification, several architectures were evaluated, including VGG19, Extreme Learning Machine (ELM), and EfficientNet-B1 and B3 combined with XGBoost. Among these, EfficientNet-B1 demonstrated superior and stable performance, achieving a test accuracy of 99.7% with very high precision across all four tumor classes.

For tumor segmentation, multiple models were compared using Dice and IoU metrics. U-Net++ with an EfficientNet-B1 encoder emerged as the best-performing model, achieving a mean Dice score of 0.9060 and a mean IoU of 0.8442. It also demonstrated strong capability in detecting small and low-contrast tumor regions, which is clinically significant for early diagnosis.

The results indicate that EfficientNet-B1-based architectures demonstrated robust performance in both classification and segmentation tasks, maintaining consistent performance across diverse datasets and supporting the feasibility of integrating such models into clinical decision-support systems to improve diagnostic efficiency.

Nevertheless, limitations remain due to variability in MRI acquisition protocols and image quality, as well as the need for model transparency and interpretability to enable clinical adoption.

Future research will focus on expanding the model to include additional types of brain tumors and their respective grades, improving advanced calibration and feature extraction strategies, extending the dataset to multi-institutional sources to enhance generalizability, and integrating hybrid learning approaches and explainability techniques to strengthen reliability and clinical applicability.

## Figures and Tables

**Table 1:** Comparison of Brain Tumor Detection Studies.

Study	Dataset	Classification	Segmentation	Accuracy/Dice
Swati et al. [1]	FigShare Brain MRI Dataset [2]	Pretrained CNN architectures (via transfer learning)	None	Prec@10 = 94.39%.
Gumaei et al. [3]	FigShare Brain MRI Dataset [2]	Regularized Extreme Learning Machine (RELM)	None	92.6144% accuracy.
Micallef et al. [4]	BraTS 2019 [5]	None	U-Net++	Dice = 0.79.
Filatov et al. [6]	Brain Tumor MRI Dataset [7]	ResNet50 EfficientNet-B1/B7 EfficientNetV2B1	None	EfficientNet-B1 with highest accuracy of 89.6%.
Gómez-Guzmán et al. [8]	Brain Tumor MRI Dataset [7]	Six pretrained CNN/transfer learning (TL) models	None	InceptionV3 with highest accuracy of 97.12%.
Poudyal et al. [10]	Brain Tumor MRI Dataset [7]	VGG16 ResNet50 DenseNet121 InceptionV3	None	DenseNet121 with highest accuracy of 94%.
Abdusalomov et al. [9]	Classification: Brain Tumor MRI Dataset [16]; Brain Tumor Classification (MRI) [17]	Yolov7	None	99.5% accuracy.
Aksoy et al. [14]	Classification: Brain Tumor MRI Dataset [16] Segmentation: BraTS [5]	Fine-tuned MobileNetV2	SegResNet	Classification: 98.09% accuracy.
Chen et al. [15]	Brain Tumor MRI Dataset [16]	Custom CNN VGGNet19 ResNet101V2 EfficientNetV2B2	None	CNN: 99.28% VGG19: 99.79% ResNet101V2: 93.88% EfficientNetV2B2: 99.01%
Xiong et al. [12]	Figshare Brain Tumor dataset [2]	Binary classification: YOLO-BT	YOLO-BT	Classification: mAP@50 = 94.7%
Gündogan [11]	Brain Tumor MRI Dataset [16]	Hybrid model combining a custom CNN + XGBoost	None	99.77% accuracy.
Valerio et al. [13]	BraTS 2023 dataset [5]	None	SegResNet	Dice score of 0.9001.

**Table 2:** Classification dataset distribution.

Class	Training Images	Testing Images	Total Images
Glioma	1147	254	1401
Meningioma	1329	306	1635
No Tumor	1067	140	1207
Pituitary	1457	300	1757
**Total**	**5000**	**1000**	**6000**

**Table 3:** Segmentation data preprocessing steps.

Step	Methods Used	Output
Data Splitting	Hold-out partitioning method	70% for train, 15% for validation, and 15% for test
Image Resizing	Area Interpolation (images), nearest-neighbor (masks)	MRI image sizes: 512 × 512 pixels for segmentation images and masks
Normalization	Min-max scaling (pixel values divided by 255.0) on images only	Normalized image pixels (0–1), masks unchanged
Data Augmentation	Random flips, rotations, brightness changes	Augmented training set with higher diversity

**Table 4:** Classification data preprocessing steps.

Step	Methods Used	Output
Data cleaning	RGB conversion	All images in RGB format
Image resizing	Area interpolation	MRI image sizes: 224 × 224 pixels
Normalization	ImageNet normalization using predefined mean [0.485, 0.456, 0.406] and standard deviation [0.229, 0.224, 0.225]	ImageNet-normalized pixel values
Label encoding	Mapping class names to numeric indices	glioma → 0, meningioma → 1, no_tumor → 2, pituitary → 3
Data splitting	Hold-out partitioning method	70% for train, 15% for validation, and 15% for test

**Table 5:** Performance Comparison of Segmentation Models.

Architecture	Train Dice	Validation Dice	Train IoU	Validation IoU
ResUNet	0.9357	0.8252	0.8798	0.7158
EfficientNet-B4	0.9163	0.8601	0.9132	0.8419
EfficientNet-B4 U-Net	0.9163	0.8716	0.8690	0.8510
Attention U-Net	0.9100	0.8695	0.8300	0.7800
U-Net 2D	0.8803	0.8512	0.8512	0.8001
U-net++	0.8884	0.8741	0.8031	0.7868

**Table 6:** Performance Metrics of Frozen EfficientNet Encoders.

Encoder	Dice	IoU
EfficientNet-B4	0.8921	0.8119
EfficientNet-B1	0.8973	0.8234

**Table 7:** Performance Metrics of Partially Fine-Tuned EfficientNet Encoders.

Encoder	Dice	IoU
EfficientNet-B4	0.8967	0.8195
EfficientNet-B1	0.9121	0.8433

**Table 8:** Segmentation test set performance.

Encoder	Dice	IoU	HD95 (Pixels)
EfficientNet-B4	0.8938	0.8304	-
EfficientNet-B1	0.9055	0.8442	12.21

**Table 9:** Performance Comparison of Classification Models.

Architecture	Train Accuracy	Validation Accuracy	Train Loss	Validation Loss
VGG19	99.80%	98.00%	0.214	0.26
ELM	89.33%	86.22%	0.40	0.58
EfficientNetV2B3 + XGBoost	100%	93.73%	0.0013	0.20
EfficientNet-B4	99.12%	99.60%	0.021	0.0011
EfficientNet-B1	100%	99.90%	0.01	0.03

**Table 10:** Precision, Recall, F1-Score, and Accuracy for Each Tumor Class.

Class	Precision	Recall	F1-Score
Glioma	1.00	1.00	1.00
Meningioma	0.99	1.00	1.00
No Tumor	0.99	1.00	1.00
Pituitary	1.00	1.00	1.00
Overall Accuracy	99.70%	–	–

**Table 11:** Accuracy Comparison of the Proposed EfficientNet-B1 Model and State-of-the-Art Methods.

Study	Dataset	Accuracy (%)
Gumaei et al. (2019) [3]	FigShare	92.61
Filatov et al. (2022) [6]	Nickparvar	89.60
Gómez-Guzmán et al. (2023) [8]	Nickparvar	97.12
Poudyal et al. (2024) [10]	Nickparvar	94.00
Abdusalomov et al. (2024) [9]	Fateh et al. [16]	99.50
Aksoy et al. (2025) [14]	Fateh et al. [16]	98.09
Chen et al. (2025)—VGG19 [15]	Fateh et al. [16]	99.79
Gündogan (2025) [11]	Fateh et al. [16]	99.77
Proposed EfficientNet-B1	BRISC2025	99.70

**Table 12:** Comparison with state-of-the-art segmentation methods.

Study	Architecture	Dice Score
Micallef et al. (2021) [4]	U-Net++	0.7900
Valerio et al. (2025) [13]	SegResNet	0.9001
**Proposed U-Net++ + EfficientNet-B1**	Proposed Model	**0.9055**

## Data Availability

The data presented in this study are available on request from the corresponding author.
